# Metallocavitins as Promising Industrial Catalysts: Recent Advances

**DOI:** 10.3389/fchem.2021.806800

**Published:** 2022-02-11

**Authors:** Albert A. Shteinman

**Affiliations:** Department of Kinetics and Catalysis, Institute of Problems of Chemical Physics, RAN, Chernogolovka, Russia

**Keywords:** metallocavitins, sustainable developing, H_2_O, CO_2_, methane

## Abstract

The energy, material, and environmental problems of society require clean materials and impose an urgent need to develop effective chemical processes for obtaining and converting energy to ensure further sustainable development. To solve these challenges, it is necessary, first of all, to learn solar energy harvesting through the development of artificial photosynthesis. In our planet, water, carbon dioxide, and methane are such affordable and inexhaustible clean materials. Electro/photocatalytic water splitting, and also CO_2_ and CH_4_ transforming into valuable products, requires the search for relevant efficient and selective processes and catalysts. Of great interest is the emerging new generation of bioinspired catalysts—metallocavitins (MCs). MCs are attracting increasing attention of researchers as advanced models of metalloenzymes, whose efficiency and selectivity are well known. The primary field of MC application is fine organic synthesis and enantioselective catalysis. On the other hand, MCs demonstrate high activity for energy challenging reactions involving small gas molecules and high selectivity for converting them into valuable products. This mini-review will highlight some recent advances in the synthesis of organic substances using MCs, but its main focus will be on the rapid development of advanced catalysts for the activation of small molecules, such as H_2_O, CO_2_, and CH_4_, and the prospects for creating related technological processes in the future.

## Introduction

**FIGURE 1:**
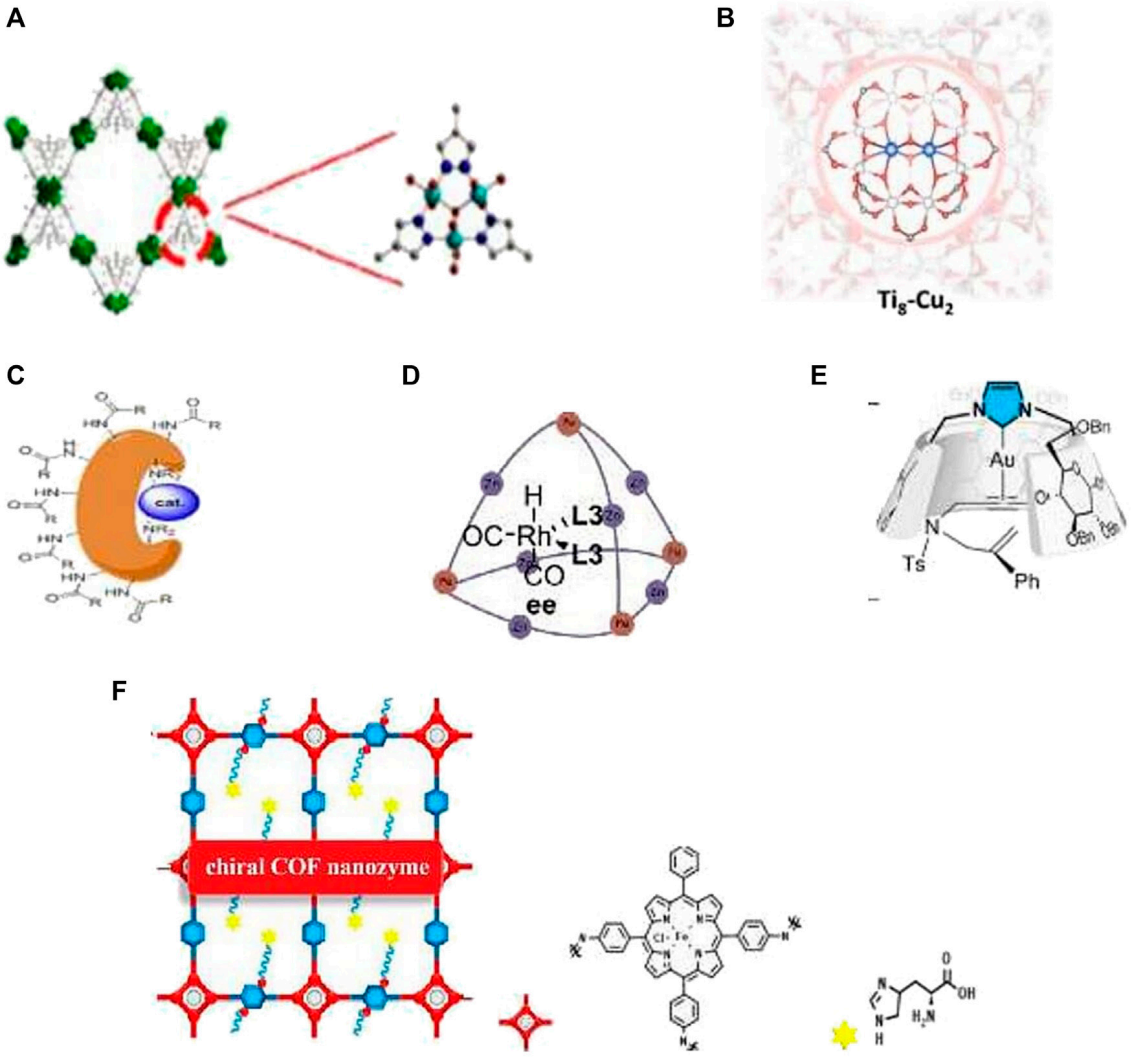
IMC as bioinspired catalysts: **(A)** Artificial catechol oxidase (Li et al, 2020). **(B)** Artificial binuclear copper monooxygenase based on Ti-MOF (Feng et al, 2021). **(C)** MC (cat-RuOs) for enantioselective dihydroxylation and epoxydation of styrene derivatives (Leurs et al, 2018). **(D)** MC for the alkene size-selective hydroformylation (Nurttila et al, 2019). **(E)** N-heterocyclic carbene-capped CD-Au complex for an enantioselective enyne cycloisomerization (Roland et al., 2018). **(F)** Chiral COF nanozyme for enantioselective L-DOPA oxidation (Zhou et al, 2020).

In recent decades, we have witnessed the emergence of an innovative generation of bioinspired catalysts—metallocavitins (MCs). Cavitins are a class of nanoporous molecules and supramolecular ensembles interesting for heterogenous metal catalysis, based usually on transition metals. The performance of the transition metal catalyst increases due to its retention in molecular nanocontainers with intrinsic porosity usually used for preparation of metallocavitins. At the same time, microporous compounds, such as charcoal or zeolites, are used long time as carriers for metal ions in industrial heterogenous catalysis because they greatly increase the performance of the encapsulated transition metals. There are two big classes of cavitins: the first one, discrete individual molecules—monocavitins, such as, for example, cyclodextrins, calixarenes, metal-organic cages (MOCs), covalent organic cages (COCs), and, the second, extended ones—polycavitins, such as zeolites, metal-organic frameworks (MOFs), covalent organic frameworks (COFs), and polypeptides, which all together demonstrate a rich library of architectures varying in shape, size, and geometry (Figure 1). MOFs and MOCs are formed by coordination-driven self-assembly. They are composed of polydentate organic linkers and inorganic nodes containing metal ions or clusters known as secondary binding units (SBUs). On the other hand, COFs and COCs are organic molecules of different sizes and complexity, synthesized *via* covalent binding. M-cavitins demonstrate high activities for energetically challenging reactions with participation of small gas molecules and high selectivity to valuable products. Nowadays, the use of new sources of clean materials and the development of efficient processes for their conversion are a necessary requirement for growing energy needs and environmental protection. In this connection, M-cavitins are promising catalysts for future technology.

## Metallocavitins as Bioinspired Catalysts

Metals in the coordination cavitins may serve either only for constructive goals or also as a coordinately unsaturated catalytic center. The general approaches for the incorporation of metal active sites into cavitins include metallolinkers, non-covalent encapsulation of metal complexes and enzymes, templated metal/ligand assemblies, and post-synthetic metalation (Leenders et al., 2015). The complexity of MOCs and COCs has increased dramatically in recent times: heteroleptic, mixed-metal, and low symmetry assemblies and different composites are becoming more commonplace (Pullen et al., 2021). The right balance between flexibility and rigidity of cavitins is favorable for binding substrates and releasing products (Zhang et al., 2017). Shape-persistent organic cages permit precise control of their size and geometry (Holsten et al., 2021). On the other hand, adaptability is a hallmark of enzymes. Flexible cavitins mimic these owing to structural changes that accompany adsorption and desorption steps (Heerden et al., 2021). Cavitins are able to create chiral molecular catalysts *via* preferential secondary interactions between the substrate and framework that induce enantioselectivities not achievable in homogenous systems. Thus, they catalyze the asymmetric acetylation of aromatic aldehydes and 2-aminobenzamide to generate the products with up to 93% yield and 97% ee (Hou et al., 2021). As was noted, the primary field of MC application is fine organic synthesis and enantioselective catalysis. It is well-known that special channels in enzymes facilitate transport of substrates and products. The mesoporous MOF MnO_2_@OMUiO-66(Ce), containing artificial substrate channels and MnO_2_ attached to Ce–O clusters, was designed as a super-active artificial catalase (Yang et al., 2021a). These authors proposed some guides for the rational design of super-active biomimetic systems. MOF-818, containing nodes of trinuclear copper centers that mimic the active sites of catechol oxidase, shows efficient catechol oxidase activity. This artificial enzyme oxidizes o-diphenoles to o-qunones with good substrate specificity (Li et al., 2020, Table 1.1, Figure 1A). The direct selective oxidations of the most difficult C–H bonds with O_2_ are very challenging reactions and play an important role in fine organic synthesis (Shteinman and Mitra, 2021). Nature has created highly active and selective binuclear metal-containing monooxygenases working with the participation of O_2_ and a reducing agent and capable of activating the most inert C–H bond of alkanes involving methane. Recently the MOF-based artificial binuclear monooxygenase Ti_8_–Cu_2_ (Table 1.2, Figure 1B) was prepared *via* metalation of the SBU in a Ti-MOF. The SBU provided a precise binding pocket for the installation of binuclear Cu cofactors to cooperatively activate O_2_. In the presence of co-reductants, Ti_8_–Cu_2_ demonstrated excellent catalytic activity and selectivity in monooxygenation processes, including epoxidation, hydroxylation, and sulfoxidation, with TOF, which is much higher than that of mononuclear Ti_8_–Cu_1_ (Feng et al., 2021). It would be interesting to check and develop this monooxygenase for alkane hydroxylation involving methane. While polycavitins are used for fabrication of advanced heterogenous catalysts (Bavykina et al., 2020), monocavitins are more suitable for modeling and academic study of the enzyme active sites. However in the recent years, MOC use in catalysis has also increased. For example, the highest activity for the enantioselective dihydroxylation and epoxidation of styrene derivatives was obtained by using Ru or Os complexes linked with bovine serum albumin (BSA, Leurs et al., 2018, Table 1.3, Figure 1C). Also, by coordination of the monocavitin, based on an Fe_4_(Zn-L)_6_ cage to rhodium, efficient hydroformylation catalysts Rh@Fe_4_(Zn-L)_6_, that shows excellent product selectivity, has been obtained (Nurttila et al, 2019; Table 1.4, Figure 1D). For the case of N-heterocyclic carbene-capped CD gold complexes (Figure 1E). (ICD)Au Cl stereoselectivity in enyne-cycloisomerization depends on the nature of the cyclodextrin: α-ICD and β- ICD give the enantiomer for which the approach is the easiest according to their helical shape, and γ- ICD does not afford enantioselectivity because of its symmetrical shape (Roland et al., 2018; Table 1.5, Figure 1E **)**. The incorporation of iron porphyrin and L- or D-histidines endows chiral COF nanozymes with high activity and selectivity in the peroxidase oxidation of dopa enantiomers (Figure 1F). This artificial peroxidase possesses 21.7 times higher activity than natural HRP (Zhou et al., 2020). “Substrate selectivity is more difficult to rationalize for small molecules, such as H_2_, O_2_, CO_2_, and CH_4_, which possess too narrow a range of physical characteristics to allow either precise positioning or discrimination between reactants. Nevertheless, metalloenzymes have evolved to metabolize these small-molecule substrates with high selectivity and efficiency due to small-molecule tunnels and gate-effects. It delivers the right substrate to the right location at the right time, for example, for selective oxidation of the strongest aliphatic hydrocarbon bond in methane” (Banerjee and Lipscomb, 2021). “Also for the small molecule discussed here, the spatial and temporal control of delivery of protons and electrons to the active site is crucial to maintain product selectivity in these transformations” (Amanullah et al., 2021). The activation of small molecules, such as CO_2_, N_2_, O_2_, and CH_4_, has always been a dream in chemistry. The modern challenges of climate change, energy deficit, and quality of the fossil resources used make the transformation of these small molecules more important than ever before. M-cavitins demonstrate high activities for energetically challenging reactions with participation of small gas molecules and high selectivity to valuable products. This explains the great interest in these processes and rapid development of advanced catalysts for the activation of small molecules, such as H_2_O, CO_2_ and CH_4_, in recent years.

**TABLE 1:** Some examples of MC catalysis of organic reactions.

Number	Reaction	MC	Yield, %	Characteristics			Figure	Reference
				TON	Selectivity,%			
1	Catechol oxidation	Cu_3_MOF-818	98	High substrate specifity			1A	Li et al. (2020)
2	Epoxydation	Cu_2_MIL-125-Ti	84–92	420–3450			1B	Feng et al. (2021)
	hydroxylation		46–94	92–480				
	sulfoxidation		95	475				
3	Epoxydation	M-BSA,(M=Ru,Os)	78	1613–2500		82(S)	1C	Leurs et al. (2018)
4	Alkene hydroformylation	RhHL_5_@Fe_4_(ZnL)_6_	33	800		99.5	1D	Nurttilla et al. (2019)
5	Enyne cycloisomerization	AuICD	99			80(+)	1E	Roland et al. (2018)

## H_2_O and Artificial Photosynthesis

**FIGURE 2:**
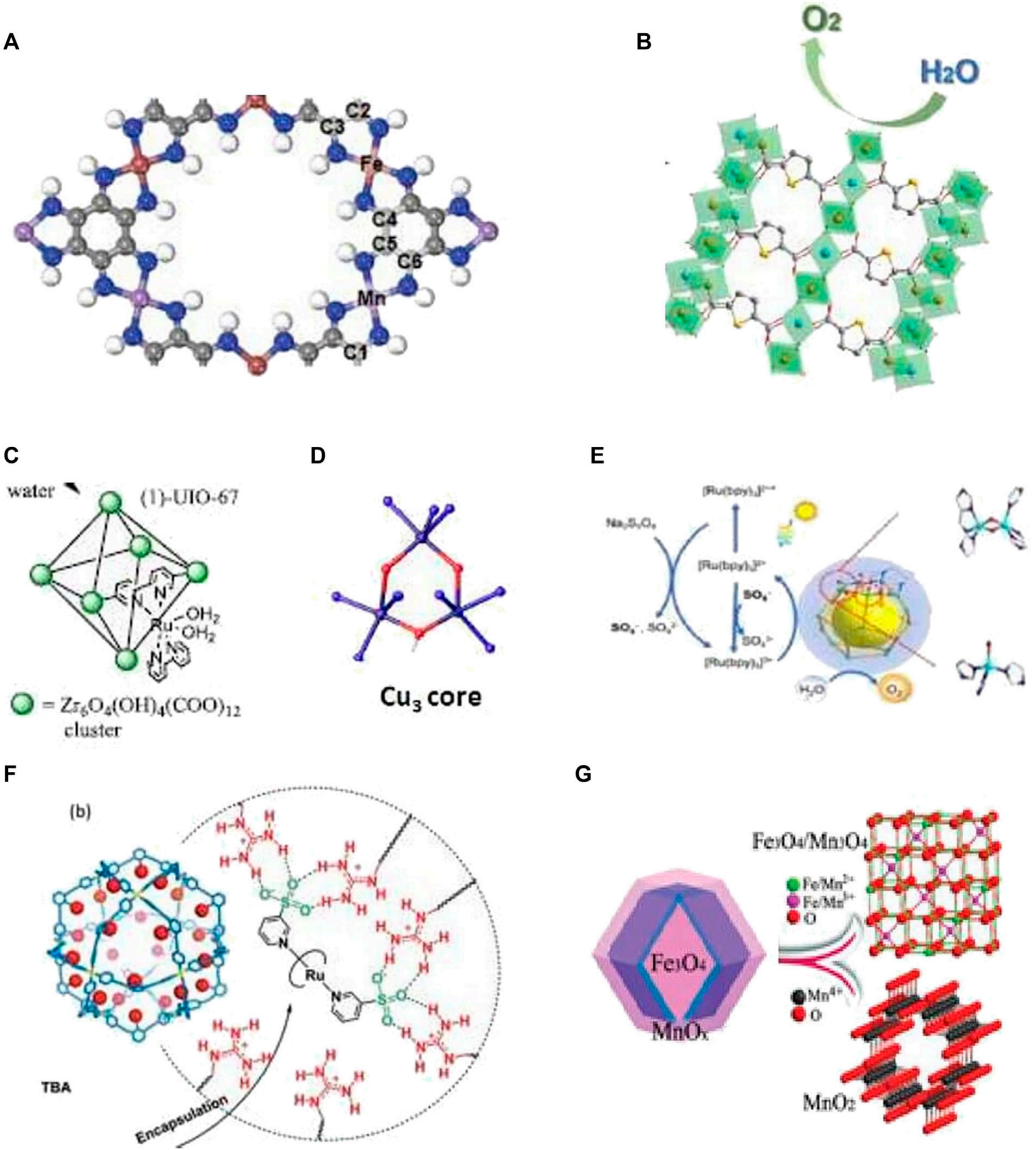
MC for WS and O reduction. **(A)** Mn/Fe-hexaiminobenzene MOF for electrocatalytic oxygen reduction reaction and OER (Shinde et al, 2019). **(B)** Fe–Ni MOF electrocatalyst for water oxidation (Wang K. et al, 2021). **(C)** Active water oxidation catalyst cis-[Ru(bpy) (5,5′ dcbpy) (H₂O)_2_]^2+^ (1) incorporated into UIO-67 MOF (Ezhov et al, 2020) **(D)** Cu-core of the bioinspired catalyst for water oxidation (Chen Z. -Y. et al, 2021). **(E)** Co-based MOC for photo-driven water oxidation (Chen Q. F. et al. 2021). **(F)** Preorganization of the ruthenium catalyst in self-assembled nanospheres for electrochemical water oxidation (Yu et al, 2018). **(G)** Hierarchical Fe–Mn binary metal oxide core-shell nano-polyhedron for electrochemical WS (Li et al, 2021).

Water in combination with sunlight can serve as a source of cheap and clean energy, provided that artificial photosynthesis is mastered. The challenges of the incumbent energy crisis and environmental problems can be solved *via* photochemical, electrochemical, and photoelectrochemical water splitting (WS) to produce oxygen and hydrogen green fuels. For the realization of artificial photosynthesis, it is “necessary to develop the design of WS catalysts that can be incorporated into future sunlight-to-chemical fuel assemblies” (Ezhov et al., 2020). WS involves two reactions: oxygen evolution reaction (OER) and hydrogen evolution reaction (HER). Photoinduced WS into oxygen and hydrogen is one of the most interesting pathways for solving the incumbent society problems. A porphyrinic zirconium MOF nanotube HNTM-Ir/Pt possessed a high photocatalytic HER rate of 201.9 mmol g^−1^h^−1^ and better recycling stability under visible light irradiation in WS than earlier HNTM-Pt or HNTM-Ir (He et al., 2018). The double-shelled TiO_2_@ZIF-8 hollow spheres used for HER under illumination show an efficient charge separation by electron injection from ZIF-8 to TiO_2_, high photocatalytic quantum efficiencies of 50.89% at 380 nm, and high HER rate up to 261.7 mmol g^−1^h^−1^, which is 3.5 times higher than that of bare TiO_2_ (Qiu et al., 2021). Donor–acceptor imine-linked COFs produce hydrogen with a rate 20.7 mmol g^−1^h^−1^ under visible light irradiation due to protonation of their imine linkages and improved charge separation efficiency (Yang et al., 2021b). Electrocatalytic WS has been regarded as another promising approach for producing hydrogen and oxygen under mild conditions. An Fe–Cu layered double hydroxide integrated with a Co MOF ZIF-12 to form LDH-ZIF-12 composite shows enhanced OER performance as compared to individual components, that is, Fe–Cu-LDH and ZIF-12. Chronoamperometric studies show that this composite leads to a higher current density and low overpotential, and is an efficient and stable electrocatalyst for commercial use (Hameed et al., 2021). The multi-shelled hollow Mn/Fe-hexaiminobenzene MOF (Mn/Fe-HIB-MOF) (Figure 2A) is an excellent bifunctional electrocatalyst for dioxygen reduction reaction and OER. It has better performance than commercial RuO_2_, Mn-HIB-MOF, and Fe-HIB-MOF catalysts (Shinde et al., 2019)]. The Fe–Ni-MOF show remarkable electrocatalytic performance with a low overpotential of 266 mV at 100 mA cm^−2^ and a high TOF value of 0.261 s^−1^ for water oxidation (Figure 2B, Wang C.-P. et al, 2021). The iridacycle-decorated COF shows 10-fold efficiency enhancement in photocatalytic hydrogen evolution from aqueous formate solution in comparison with its molecular counterpart under mild conditions (Hu et al., 2021). Photoinduced water oxidation by MOFs has been widely studied in the past few years. The active water oxidation catalyst *cis*-[Ru(bpy) (5,5′-dcbpy) (H_2_O)_2_]^2+^ was incorporated into UIO-67 MOFs using postsynthetic modification of the framework (Figure 2C). XAS, EPR, and Raman spectroscopy confirmed the formation of M-cavitin and the highly active Ru^V^ = O key intermediate (Ezhov et al., 2020). A bioinspired trinuclear copper catalyst developed recently for water oxidation displays a TOF value of 20 000 s^−1^, which is 150 times and 15 times higher than that of the mono- and the bis-Cu complexes, respectively (Figure 2D; Chen Q.-F. et al, 2021). Also, four MOCs based on cobalt ions and imidazolate ligands were studied recently on photo-driven water oxidation for the first time (Chen Z.-Y. et al, 2021). These studies revealed that photoinduced water oxidation starts *via* e^−^ transfer from the excited [Ru(bpy)_3_]^2+^* to Na_2_S_2_O_8_ and then to the bis (μ-oxo) dicobalt active sites which further donate electrons to the oxidized [Ru(bpy)_3_]^3+^ to drive water oxidation (Figure 2E). Self-assembled nanospheres with guanidinium binding sites form sulfonate-functionalized preorganized ruthenium catalysts for electrochemical water oxidation, which leads to an increase in the reaction rate by almost 100 times compared to a homogenous system (Figure 2F) (Yu et al., 2018). Hierarchical bifunctional catalysts for WS are undoubtedly the most promising catalysts of the low-carbon energy future. Such a Fe_3_O_4_@MnO_x_ binary metal oxide core–shell nano-polyhedron fabricated recently (Figure 2F, Li et al., 2021) involves cathodic 2e^−^ HER and anodic 4 e^−^ OER and has a low HER/OER overpotential of 242/188 mV (10 mA cm^−2^), a small Tafel slope of 116.4/77.6 mV dec^−1,^ and a long-term cyclability of 5 h. By applying this catalyst as an independent cathode and anode, the overall WS cell supplies a competitive voltage of 1.64 V to achieve 10 mA cm^−2^ and good work stability of 80 h. Another example is Fe-doped NiCo_2_O_4_/Ni_3_S_4_ hollow heterostructure nanotubes which implement highly efficient electrocatalytic overall WS with low overpotential of 29.1 mV (10 mA cm^−2^), a relatively small Tafel slope of 53.3 mV dec^−1^ for the HER, and 259 mV at a current density of 100 mA cm^−2^ (33.1 mV dec^−1^) for the OER (Liu et al., 2021).

## CO_2_ and Methane

**FIGURE 3:**
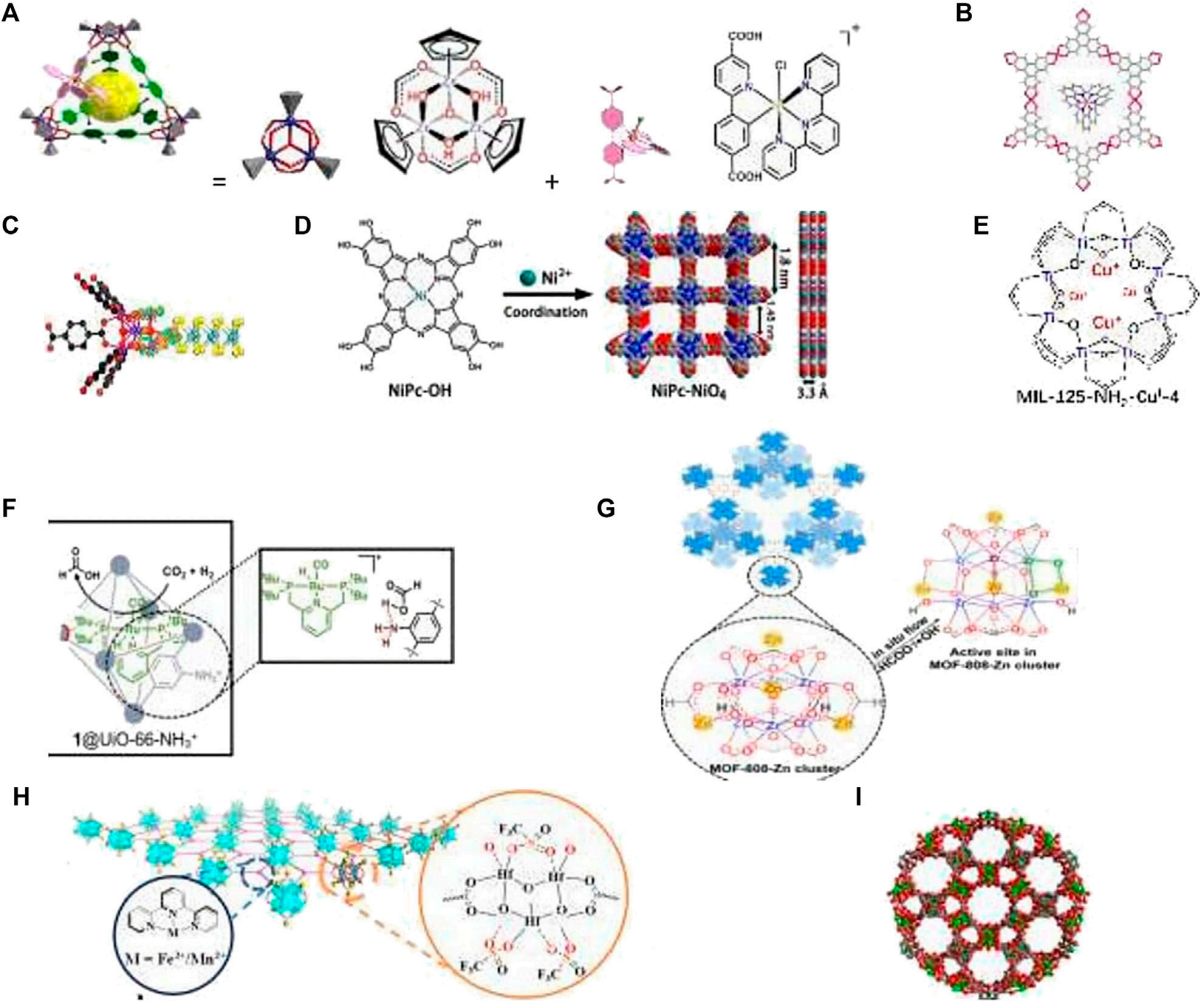
MC for CO_2_ transformations: **(A)** MOC decorated with an Ir(III) complex for CO_2_ photoreduction. **(B)** Electrostatic attraction-driven assembly of MOF with a photosensitizer for photocatalytic CO_2_ reduction to CO (Huang et al, 2021). **(C)** Hierarchically porous d-UiO-66/MoS_2_ for selective conversion of CO_2_ with 1–120 into CH_3_COOH (Yu T. et al, 2021). **(D)** Conductive two-dimensional phthalocyanine-based MOF nanosheets linked by nickel-catecholate for CO_2_ electroreduction. **(E)** Multiple cuprous centers supported on Ti-MOF for CO_2_ hydrogenation to ethylene (Zeng et al, 2021). **(F)** Multicomponent catalyst system based on MOF UiO-66 for the hydrogenation of carbon dioxide to methanol (Rayder et al, 2021). **(G)** MOF-808-Zn catalyst for CO_2_ hydrogenation (Zhang H. et al, 2021). **(H)** Bifunctional Metal-Organic Layers for tandem catalytic transformations using molecular oxygen and carbon dioxide (Shi et al, 2021). **(I)** COF assembled from 12-nuclear [Cu^12^] nanocages with two types nanotubular channels for a catalysis the cycloaddition of CO_2_ to various epoxides (Wang K. et al, 2021).

Mastering artificial photosynthesis includes along with photolytic WS also solar light harvesting and photochemical reduction of carbon dioxide (CO_2_R). The light harvesting systems based on supramolecular assembly in aqueous media contain light-absorbing chromophores which gather and convert the excitation energy to chemical energy *via* energy transfers from donors to acceptors. Some cavitins, for example, hydrophobic macrocycles, such as CD and calixarenes, play a prominent role in forming these supramolecular systems (Wang H et al., 2021). The photochemical CO_2_R was studied for a number of MCs. In cavitins, the transition state of the target reaction can be stabilized more efficiently in comparison with bulk solution. Thus, Ir complex incorporated into Zr-MOC-NH_2_ was used for CO_2_ photoreduction. DFT calculations and *in situ* IR show that the Ir(III) complex is the catalytic center and −NH_2_ in the cavity plays a synergetic role in the stabilization of the transition state and Ir∙CO_2_ intermediate (Qi et al., 2021). Under irradiation by visible light, the single Ir^III^-MOC-NH_2_ cage can convert CO_2_ into CO with a selectivity of 99.5% and a TOF of 120 h^−1^, which is 3.4 times higher that of bulk Ir(III) complex and 100 times higher than that of the classical MOF counterpart, Ir^III^-Uio-67-NH_2_ (Figure 3A, Qi et al., 2021). Photocatalytic CO_2_ R with the MIL-100(Fe)-CsPbBr_3_ composite having a high specific surface area, an enhanced solar light response, and an improved charge carrier separation resulted in an excellent photocatalytic performance with 20.4 μmol CO produced per gram of the photocatalyst during 1 hour under visible light irradiation (Cheng et al., 2020). Anionic MOFs as a host and a cationic photosensitizer as a guest were self-assembled into a photocatalytic system, Ru@Cu-HHTP, which showed high activity for photocatalytic CO_2_R under sunlight with CO selectivity of 91.3% (Figure 3B, Huang et al., 2021). An Ni-MOF–derived catalyst for the light-driven methanation of CO_2_ produces methane with a rate of 488 mmol g^_1^h^_1^ under UV–visible–IR irradiation and displayed excellent recyclability without loss of catalytic activity (Khan et al., 2021). Because of the highly stacked layers, some COFs have semiconductive properties and exhibit good catalytic performance in photo-CO_2_R (Nguyen and Alzamly, 2021). The d-UiO-66/MoS_2_ composite implements the photocatalytic conversion of CO_2_ and H_2_O to CH_3_COOH under visible light. The evolution rate and selectivity of CH_3_COOH reached 39.0 mmol g^_1^h^_1^ and 94%, respectively, without any C1 products (Figure 3C, Yu F. et al, 2021). Electrochemical CO_2_R has also been proven to be a promising strategy among various types of energy conversion. A desired electrocatalyst should have a high TON, a high TOF, and low overpotential (Zhang H. et al, 2021). The Zn-based MOF CALF20 demonstrates the highest CO Faradaic efficiency of 94.5% at −0.97 V *vs*. RHE with a TOF of 1360.8 h^−1^ and a partial current density of −32.8 mA/cm^2^ for electrochemical CO_2_R to CO (Al-Attas et al., 2021). The redox-active In-MOF shows the first example of an Ni-based MOF catalyst in electrocatalytic CO_2_R, which suggests new prospects for designing novel and efficient non-noble, metal-based, redox-active, biomimetic MOFs (Zhou et al., 2021). Conductive phthalocyanine-based MOF (NiPc-NiO_4_) nanosheets linked by nickel-catecholate are highly efficient electrocatalysts for the CO_2_R to CO electroreduction (Figure 3D). The obtained NiPc–NiO_4_ has good conductivity and exhibited a selectivity of 98.4% toward CO production and a large CO partial current density of 34.5 mA cm^−2^, outperforming the reported MOF catalysts (Yi et al., 2021). Hydrogenation of CO_2_ to valuable chemicals may also be of great interest for the implementation of a sustainable carbon cycle. Multiple Cu centers supported on Ti-MOF **(** Figure 3E **)** catalyze CO_2_ hydrogenation to ethylene. Zeng et al., (2021) present a new tandem route for CO_2_-to-C_2_H_4_ conversion *via* CO_2_ hydrogenation to ethanol followed by its dehydration. The catalyst exhibits high ethylene selectivity, that is, > 90%. The MOF UiO-66 was used in tandem with its zirconium oxide nodes and an incorporated ruthenium PNP pincer complex to hydrogenate CO_2_ to HCOOH, then to CH_3_OH, giving the highest reported TON 19 000 and TOF 9100 h^−1^. Moreover, the reaction was readily recyclable, leading to a cumulative TON of 100,000 after 10 reaction cycles (Figure 3F, Rayder et al., 2021). The neighboring Zn^2+^-O-Zr^4+^ sites obtained by postsynthetic treatment of Zr_6_(μ_3_-O)_4_(μ_3_-OH)_4_ nodes of MOF-808 by ZnEt_2_ gave the MOF-808-Zn catalyst, which exhibits >99% MeOH selectivity in CO_2_ hydrogenation at 250°C and a high space-time yield of up to 190.7 mg _MeOH_ g_Zn_^−1^ h^−1^ and exhibited excellent stability for at least 100 h (Figure 3G, Zhang Y. et al, 2021). Bifunctional MOF containing tripyridyl complexes of Fe and Mn converts styrenes into styrene carbonates *via* tandem epoxydation using O_2_ and then CO_2_ insertion and also effectively transforms alkylaromatic hydrocarbons into cyanohydrins via involvement of a high-spin Fe^IV^ (S = 2) center in the challenging oxidation of the sp3 C–H bond. (Figure 3H, Shi et al., 2021). A new porous COF assembled from 12-nuclear [Cu_12_] nanocages {[Cu_2_(L^4−^) (H_2_O)_2_]·4DMA·2H_2_O}_n_ with two types of nanotubular channels and a large specific surface area effectively catalyzes the cycloaddition of CO_2_ to various epoxides under mild conditions (Figure 3I, Wang W.-M. et al, 2021). Carbonylation reactions are of particular importance for organic synthesis. The use of CO_2_ in the carbonylation reaction catalyzed by composite (Cu_1_Pd_2_)_z_@PCN-222(Co) allows the photosynthesis of benzophenone with 90% yield and 97% selectivity under mild conditions (Fu et al., 2021). Cavitins are of interest in the development of tandem methylation reactions utilizing CO_2_ as a one-carbon building block that would enable a more sustainable chemical industry (Rooney et al., 2021). The development of the direct low-temperature selective oxidation of methane to methanol has remained an active area of research over the last 50 years (Shteinman, 2020). Compared to WS and CO_2_ conversion, the effective and selective chemical routes to valorize methane with participation of cavitins are relatively less elaborated. Direct methane conversion has been carried out in the gas phase over Cu- and Fe-containing zeolites in the stepwise cyclic process that involves activating the transition metal with O_2_ or N_2_O at 400–500^o^ C, then methane reaction with active intermediates at 200^o^ C, and finally product extraction with water steam. However, the rate and productivity of these processes are still very low (Shteinman, 2020). Catalytic processes for the hydroxylation of methane on zeolite Fe-Cu-ZSM-5 with a selectivity of 20–80% under aqueous conditions at 50°C using H_2_O_2_ were proposed (Hammond et al., 2012; Yu T. et al, 2021). The Cu–Fe (2/0.1)/ZSM-5 catalyst is an efficient catalyst for the direct conversion of methane into methanol in the liquid phase using H_2_O_2_, which exhibits an excellent methanol productivity of 431 mol_MeOH_·mol^−1^_Fe_·h^−1^ and methanol selectivity of 80%. The mechanism based on catalytic, spectroscopic, and theoretical results was suggested (Yu T. et al, 2021): the acid sites adjacent to iron Bronsted contribute to the formation of an active Fe (V) = O intermediate *via* the dehydration of formed Fe−OOH in the aqueous H_2_O_2_ solution, enabling the homolytic cleavage of the primary C−H by radical-rebound mechanism to generate •CH_3_ radicals that are quickly captured by •OH radicals to form CH_3_OH. Contrary to Cu- and Fe-containing zeolites, the study of MOF-based MMO mimics is still at the early stage and suffers from the same problems which are low productivity and rate and low methanol selectivity due to overoxidation (Shteinman, 2020).

## Summary, Discussion, and Perspective

Cavities and other holes are ubiquitous in the material world. Enzymes are inherently natural cavitins. Chemical cavitins have emerged due to efforts of synthetic and supramolecular chemists. M-cavitins demonstrate high activities for energetically challenging reactions. Much information has already been accumulated on the photocatalytic and electrochemical conversion of energy for the ecological production of fuels, materials, and chemicals. Still, looking ahead, innovative solutions are still needed to overcome the global energy crisis and improve the environment. The solution of these problems is primarily connected with the development of fundamental scientific research of bioinspired catalysts. These studies will require completely new solutions for the design and synthesis of a more diverse library of M-cavitins with innovative structures, metal ion composition, and functionality. Despite many achievements of electrocatalytic WS, highly active and durable catalysts have to be developed to overcome the kinetic barriers in this process, especially for the OER (Zhang H. et al, 2021). Electrocatalysis of other abundant resources, such as methane, is now increasingly coming into focus. In this area much research remains to be carried out. In particular, efforts to delineate reaction mechanisms and extract the fundamental insights are necessary to develop economically competitive electrosynthetic routes using methane. Though there still remains a considerable gap between academic research and industrial applicability, the fundamental research covered here with atomically precise catalytic systems serves as a promising foundation for the future (Zhang H et al., 2021). Using visible light irradiation to reduce CO_2_ to C-based products is an environmental and economic method which transforms solar energy in the form of chemical bonds. COF chemistry has been exponentially explored during the last several years, and the photocatalytic CO_2_ R processes catalyzed by COF materials are in their early stage (Nguyen and Alzamly, 2021). The direct oxidation of methane in a laboratory setup with participation of M-cavitins using mild conditions is still a challenging problem. There is still a huge gap between MMO enzymes and chemical catalysts based on M-cavitins both in activity and selectivity and also, importantly, in the mechanism of direct oxidation of methane to methanol.

## References

[B1] Al-Attas T. A. Marei N. N. Yong X. Yasri N. G. Thangadurai V. Shimizu G. (2021). Ligand-Engineered Metal-Organic Frameworks for Electrochemical Reduction of Carbon Dioxide to Carbon Monoxide. ACS Catal. 11, 7350–7357. 10.1021/acscatal.1c01506

[B2] Amanullah S. Saha P. Nayek A. Ahmed M. E. Dey A. (2021). Biochemical and Artificial Pathways for the Reduction of Carbon Dioxide, Nitrite and the Competing Proton Reduction: Effect of 2nd Sphere Interactions in Catalysis. Chem. Soc. Rev. 50, 3755–3823. 10.1039/d0cs01405b 33514959

[B3] Banerjee R. Lipscomb J. D. (2021). Small-Molecule Tunnels in Metalloenzymes Viewed as Extensions of the Active Site. Acc. Chem. Res. 54, 2185–2195. 10.1021/acs.accounts.1c00058 33886257 PMC8130187

[B4] Bavykina A. Kolobov N. Khan I. S. Bau J. A. Ramirez A. Gascon J. (2020). Metal-Organic Frameworks in Heterogeneous Catalysis: Recent Progress, New Trends, and Future Perspectives. Chem. Rev. 120 (16), 8468–8535. 10.1021/acs.chemrev.9b00685 32223183

[B5] Chen Q.-F. Cheng Z.-Y. Liao R.-Z. Zhang M.-T. (2021). Bioinspired Trinuclear Copper Catalyst for Water Oxidation with a Turnover Frequency up to 20000 S-1. J. Am. Chem. Soc. 143, 19761–19768. 10.1021/jacs.1c08078 34793144

[B6] Chen Z.-Y. Long Z.-H. Wang X.-Z. Zhou J.-Y. Wang X.-S. Zhou X.-P. (2021). Cobalt-Based Metal-Organic Cages for Visible-Light-Driven Water Oxidation. Inorg. Chem. 60, 10380–10386. 10.1021/acs.inorgchem.1c00907 34171190

[B7] Cheng R. Debroye E. Hofkens J. Roeffaers M. B. J. (2020). Efficient Photocatalytic CO_2_ Reduction with MIL-100(Fe)-CsPbBr_3_ Composites. Catalysts 10, 1352. 10.3390/catal10111352

[B8] Ezhov R. Karbakhsh Ravari A. Page A. Pushkar Y. (2020). Water Oxidation Catalyst Cis-[Ru(bpy)(5,5′-dcbpy)(H_2_O)2]^2+^ and Its Stabilization in Metal-Organic Framework. ACS Catal. 10, 5299–5308. 10.1021/acscatal.0c00488

[B9] Feng X. Song Y. Chen J. S. Xu Z. Dunn S. J. Lin W. (2021). Rational Construction of an Artificial Binuclear Copper Monooxygenase in a Metal-Organic Framework. J. Am. Chem. Soc. 143, 1107–1118. 10.1021/jacs.0c11920 33411525

[B10] Fu S. Yao S. Guo S. Guo G.-C. Yuan W. Lu T.-B. (2021). Feeding Carbonylation with CO_2_ via the Synergy of Single-Site/Nanocluster Catalysts in a Photosensitizing MOF. J. Am. Chem. Soc. 143, 20792–20801. 10.1021/jacs.1c08908 34865490

[B11] Hameed A. Batool M. Iqbal W. Abbas S. Imran M. Khan I. A. (2021). ZIF-12/Fe-Cu LDH Composite as a High Performance Electrocatalyst for Water Oxidation. Front. Chem. 9, 686968. 10.3389/fchem.2021.686968 34249860 PMC8264502

[B12] Hammond C. Forde M. M. Ab Rahim M. H. Thetford A. He Q. Jenkins R. L. (2012). Direct Catalytic Conversion of Methane to Methanol in an Aqueous Medium by Using Copper-Promoted Fe-ZSM-5. Angew. Chem. Int. Ed. 51, 5129–5133. 10.1002/anie.201108706 22488717

[B13] He T. Chen S. Ni B. Gong Y. Wu Z. Song L. (2018). Zirconium-Porphyrin-Based Metal-Organic Framework Hollow Nanotubes for Immobilization of Noble-Metal Single Atoms. Angew. Chem. Int. Ed. 57, 3493–3498. 10.1002/anie.201800817 29380509

[B14] Heerden D. P. Smith V. J. Aggarwal H. Barbour L. J. (2021). High Pressure *In Situ* Single‐Crystal X‐Ray Diffraction Reveals Turnstile Linker Rotation Upon Room‐Temperature Stepped Uptake of Alkanes. Angew. Chem. Int. Ed. 60, 13430–13435. 10.1002/anie.202102327 33780117

[B15] Holsten M. Feierabend S. Elbert S. M. Rominger F. Oeser T. Mastalerz M. (2021). Soluble Congeners of Prior Insoluble Shape Persistent Imine Cages. Chem. Eur. J. 27, 9383–9390. 10.1002/chem.202100666 33848032 PMC8362185

[B16] Hou B. Yang S. Yang K. Han X. Tang X. Liu Y. (2021). Confinement‐Driven Enantioselectivity in 3D Porous Chiral Covalent Organic Frameworks. Angew. Chem. Int. Ed. 60, 6086–6093. 10.1002/anie.202013926 33295124

[B17] Hu J. Mehrabi H. Meng Y.-S. Taylor M. Zhan J.-H. Yan Q. (2021). Probe Metal Binding Mode of Imine Covalent Organic Frameworks: Cycloiridation for (Photo)catalytic Hydrogen Evolution from Formate. Chem. Sci. 12, 7930–7936. 10.1039/d1sc01692j 34168847 PMC8188469

[B18] Huang N.-Y. He H. Liu S. Zhu H.-L. Li Y.-J. Xu J. (2021). Electrostatic Attraction-Driven Assembly of a Metal-Organic Framework with a Photosensitizer Boosts Photocatalytic CO_2_ Reduction to CO. J. Am. Chem. Soc. 143, 17424–17430. 10.1021/jacs.1c05839 34637290

[B19] Khan I. S. Mateo D. Shterk G. Shoinkhorova T. Poloneeva D. Garzón Tovar L. (2021). An Efficient Metal-Organic Framework Derived Nickel Catalyst for the Light Driven Methanation of CO_2_ . Angew. Chem. Int. Ed. 60, 26476–26482. 10.1002/anie.202111854 34648675

[B20] Leenders S. H. A. M. Gramage-Doria R. de Bruin B. Reek J. N. H. (2015). Transition Metal Catalysis in Confined Spaces. Chem. Soc. Rev. 44, 433–448. 10.1039/c4cs00192c 25340992

[B21] Leurs M. Dorn B. Wilhelm S. Manisegaran M. Tiller J. C. (2018). Multicore Artificial Metalloenzymes Derived from Acylated Proteins as Catalysts for the Enantioselective Dihydroxylation and Epoxidation of Styrene Derivatives. Chem. Eur. J. 24, 10859–10867. 10.1002/chem.201802185 29808506

[B22] Li M. Chen J. Wu W. Fang Y. Dong S. (2020). Oxidase-like MOF-818 Nanozyme with High Specificity for Catalysis of Catechol Oxidation. J. Am. Chem. Soc. 142, 15569–15574. 10.1021/jacs.0c07273 32790301

[B23] Li Y.-W. Su S.-K. Yue C.-Z. Shu J. Zhang P.-F. Du F.-H. (2021). Hierarchical Fe-Mn Binary Metal Oxide Core-Shell Nano-Polyhedron as a Bifunctional Electrocatalyst for Efficient Water Splitting. Dalton Trans. 50, 17265–17274. 10.1039/d1dt03048e 34787163

[B24] Liu Z. Zhao B. Pan C. Zhao H. (2021). Binder-free Fe-Doped NiCo2O4/Ni3S4 Hollow Heterostructure Nanotubes for Highly Efficient Overall Water Splitting. Dalton Trans. 50, 18155–18163. 10.1039/d1dt02904e 34854866

[B25] Nguyen H. L. Alzamly A. (2021). Covalent Organic Frameworks as Emerging Platforms for CO_2_ Photoreduction. ACS Catal. 11, 9809–9824. 10.1021/acscatal.1c02459

[B51] Nurttila S. S. Brenner W. Mosquera J. van Vliet K. M. Nitschke J. R. Reek J. N. H. (2019). Size-Selective Hydroformylation by a Rhodium Catalyst Confined in a Supramolecular Cage. Chem. Eur. J. 25, 609–620. 10.1002/chem.201804333 30351486 PMC6391983

[B26] Pullen S. Tessarolo J. Clever G. H. (2021). Increasing Structural and Functional Complexity in Self-Assembled Coordination Cages. Chem. Sci. 12, 7269–7293. 10.1039/d1sc01226f 34163819 PMC8171321

[B27] Qi X. Zhong R. Chen M. Sun C. You S. Gu J. (2021). Single Metal-Organic Cage Decorated with an Ir(III) Complex for CO_2_ Photoreduction. ACS Catal. 11, 7241–7248. 10.1021/acscatal.1c01974

[B28] Qiu T. Gao S. Liang Z. Wang D. G. Tabassum H. Zhong R. (2021). Pristine Hollow Metal-Organic Frameworks: Design, Synthesis and Application. Angew. Chem. Int. Ed. 60, 17314–17336. 10.1002/anie.202012699 33124724

[B29] Rayder T. M. Bensalah A. T. Li B. Byers J. A. Tsung C.-K. (2021). Engineering Second Sphere Interactions in a Host-Guest Multicomponent Catalyst System for the Hydrogenation of Carbon Dioxide to Methanol. J. Am. Chem. Soc. 143, 1630–1640. 10.1021/jacs.0c08957 33464883

[B52] Roland S. Suarez J. M. M. Sollogoub M. (2018). Confinement of Metal–N-Heterocyclic Carbene Complexes to Control Reactivity in Catalytic Reactions. Chem. Eur. J. 24, 12464–12473. 10.1002/chem.201801278 29617045

[B30] Rooney C. L. Wu Y. Tao Z. Wang H. (2021). Electrochemical Reductive N-Methylation with CO_2_ Enabled by a Molecular Catalyst. J. Am. Chem. Soc. 143, 19983–19991. 10.1021/jacs.1c10863 34784216

[B31] Shi W. Quan Y. Lan G. Ni K. Song Y. Jiang X. (2021). Bifunctional Metal-Organic Layers for Tandem Catalytic Transformations Using Molecular Oxygen and Carbon Dioxide. J. Am. Chem. Soc. 143, 16718–16724. 10.1021/jacs.1c07963 34592814

[B32] Shinde S. S. Lee C. H. Jung J.-Y. Wagh N. K. Kim S.-H. Kim D.-H. (2019). Unveiling Dual-Linkage 3D Hexaiminobenzene Metal-Organic Frameworks towards Long-Lasting Advanced Reversible Zn-Air Batteries. Energy Environ. Sci. 12, 727–738. 10.1039/C8EE02679C

[B33] Shteinman A. A. (2020). Bioinspired Oxidation of Methane: from Academic Models of Methane Monooxygenases to Direct Conversion of Methane to Methanol. Kinet Catal. 61, 339–359. 10.1134/S0023158420030180

[B34] Shteinman A. A. Mitra M. (2021). Nonheme Mono- and Dinuclear Iron Complexes in Bio-Inspired C-H and C-C Bond Hydroxylation Reactions: Mechanistic Insight. Inorg. Chim. Acta 523, 120388. 10.1016/j.ica.2021.120388

[B35] Wang C.-P. Feng Y. Sun H. Wang Y. Yin J. Yao Z. (2021). Self-Optimized Metal-Organic Framework Electrocatalysts with Structural Stability and High Current Tolerance for Water Oxidation. ACS Catal. 11, 7132–7143. 10.1021/acscatal.1c01447

[B36] Wang H. Jin Y. Sun N. Zhang W. Jiang J. (2021). Post-synthetic Modification of Porous Organic Cages. Chem. Soc. Rev. 50, 8874–8886. 10.1039/d0cs01142h 34180920

[B37] Wang K. Velmurugan K. Li B. Hu X.-Y. (2021). Artificial Light-Harvesting Systems Based on Macrocycle-Assisted Supramolecular Assembly in Aqueous media. Chem. Commun. 57, 13641–13654. 10.1039/d1cc06011b 34871337

[B38] Wang W.-M. Wang W.-T. Wang M.-Y. Gu A.-L. Hu T.-D. Zhang Y.-X. (2021). A Porous Copper-Organic Framework Assembled by [Cu_12_] Nanocages: Highly Efficient CO_2_ Capture and Chemical Fixation and Theoretical DFT Calculations. Inorg. Chem. 60, 9122–9131. 10.1021/acs.inorgchem.1c01104 34061517

[B39] Yang J. Li K. Li C. Gu J. (2021a). *In Situ* Coupling of Catalytic Centers into Artificial Substrate Mesochannels as Super‐Active Metalloenzyme Mimics. Small 17 (35), 2101455. 10.1002/smll.202101455 34310077

[B40] Yang J. Acharjya A. Ye M. Y. Rabeah J. Li S. Kochovski Z. (2021b). Protonated Imine‐Linked Covalent Organic Frameworks for Photocatalytic Hydrogen Evolution. Angew. Chem. Int. Ed. 60, 19797–19803. 10.1002/anie.202104870 PMC8457210 34043858

[B41] Yi J. D. Si D. H. Xie R. Yin Q. Zhang M. D. Wu Q. (2021). Conductive Two‐Dimensional Phthalocyanine‐based Metal-Organic Framework Nanosheets for Efficient Electroreduction of CO_2_ . Angew. Chem. Int. Ed. 60, 17108–17114. 10.1002/anie.202104564 34033203

[B42] Yu F. Jing X. Wang Y. Sun M. Duan C. (2021). Hierarchically Porous Metal-Organic Framework/MoS_2_ Interface for Selective Photocatalytic Conversion of CO_2_ with H_2_O into CH_3_COOH. Angew. Chem. Int. Ed. 60, 24849–24853. 10.1002/anie.202108892 34435428

[B43] Yu F. Poole D. Mathew S. Yan N. Hessels J. Orth N. (2018). Control over Electrochemical Water Oxidation Catalysis by Preorganization of Molecular Ruthenium Catalysts in Self-Assembled Nanospheres. Angew. Chem. Int. Ed. 57, 11247–11251. 10.1002/anie.201805244 PMC6120458 29975448

[B44] Yu T. Li Z. Lin L. Chu S. Su Y. Song W. (2021). Highly Selective Oxidation of Methane into Methanol over Cu-Promoted Monomeric Fe/ZSM-5. ACS Catal. 11, 6684–6691. 10.1021/acscatal.1c00905

[B45] Zeng L. Cao Y. Li Z. Dai Y. Wang Y. An B. (2021). Multiple Cuprous Centers Supported on a Titanium-Based Metal-Organic Framework Catalyze CO_2_ Hydrogenation to Ethylene. ACS Catal. 11, 11696–11705. 10.1021/acscatal.1c01939

[B46] Zhang D. Martinez A. Dutasta J.-P. (2017). Emergence of Hemicryptophanes: From Synthesis to Applications for Recognition, Molecular Machines, and Supramolecular Catalysis. Chem. Rev. 117, 4900–4942. 10.1021/acs.chemrev.6b00847 28277650

[B47] Zhang H. Cheng W. Luan D. Lou X. W. (2021). Atomically Dispersed Reactive Centers for Electrocatalytic CO_2_ Reduction and Water Splitting. Angew. Chem. Int. Ed. 60, 13177–13196. 10.1002/anie.202014112 PMC8248387 33314631

[B48] Zhang Y. Li J. Kornienko N. (2021). Towards Atomic Precision in HMF and Methane Oxidation Electrocatalysts. Chem. Commun. 57, 4230–4238. 10.1039/d1cc01155c 33861272

[B49] Zhou Y. Liu S. Gu Y. Wen G.-H. Ma J. Zuo J.-L. (2021). In(III) Metal-Organic Framework Incorporated with Enzyme-Mimicking Nickel Bis(dithiolene) Ligand for Highly Selective CO_2_ Electroreduction. J. Am. Chem. Soc. 143, 14071–14076. 10.1021/jacs.1c06797 34450022

[B50] Zhou Y. Wei Y. Ren J. Qu X. (2020). A Chiral Covalent Organic Frameworks (COFs) Nanozyme with Ultrahigh Enzymatic Activity. Mater. Horiz. 7, 3291–3297. 10.1039/D0MH01535K

